# Development and validation of a prediction model for knee joint line orientation after high tibial osteotomy

**DOI:** 10.1186/s12891-019-2820-9

**Published:** 2019-09-17

**Authors:** Jae-Young Park, Chong Bum Chang, Dong-Wan Kang, Sohee Oh, Seung-Baik Kang, Myung Chul Lee

**Affiliations:** 1 Department of Orthopaedic Surgery, Kyung Hee University Hospital, Seoul, South Korea; 2 Department of Orthopaedic Surgery, Seoul National University College of Medicine, Seoul, South Korea; 3 Department of Orthopaedic Surgery, Seoul National University Bundang Hospital, 82, Gumi-ro 173 Beon-gil, Bundang-gu, Seongnam-si, Gyeonggi-do 13620 South Korea; 4 Department of Orthopaedic Surgery, Seoul National University Hospital, Seoul, South Korea; 5 Department of Biostatistics, SMG-SNU Boramae Medical Center, Seoul, South Korea; 6 Department of Orthopaedic Surgery, SMG-SNU Boramae Medical Center, Seoul, South Korea

**Keywords:** High tibial osteotomy, Knee joint line orientation

## Abstract

**Background:**

Maintenance of optimal knee joint line orientation (KJLO) is important after high tibial osteotomy (HTO). No tools, however, are currently available that could predict the value of postoperative KJLO before surgery. First, this study sought to determine the effects of various preoperative anatomical alignment parameters to postoperative KJLO. Based upon these analyses, we aimed to devise an equation that predicts the value of postoperative KJLO.

**Methods:**

A total of 14 radiographic parameters were measured in preoperative and postoperative full-limb standing anteroposterior radiographs on 50 patients who underwent open-wedge HTO. The parameters were analysed using multivariable linear regression to predict KJLO after HTO. External validation of the equation was done with 20 patients who underwent HTO at another institution.

**Results:**

After HTO, KJLO increased from − 0.8° to 2.9° (*P* < 0.001). Based on the multivariable linear regression analysis, an equation was derived that can estimate postoperative KJLO after HTO; postoperative KJLO(°) = 1.029 + 0.560 × preoperative KJLO(°) + 0.310 × preoperative tibia plateau inclination(°) + 0.463 × aimed correction angle(°). The adjusted coefficients of determination value for this equation was 0.721. The equation also showed good calibration and predictability in external validation with predicted squared correlation coefficient of 0.867.

**Conclusions:**

This study analysed the effects of preoperative anatomical alignment parameters on the postoperative KJLO. An equation which predicts postoperative KJLO with preoperative anatomical alignment factors was devised and validated. This equation would help in selecting optimal patients for HTO and in selecting the optimal target correction angle in HTO.

## Introduction

High tibial osteotomy (HTO) is a common realignment operation for the treatment of medial compartment osteoarthritis (OA) with varus malalignment of the knee joint. Medial open-wedge high tibial osteotomy (OWHTO) has gained popularity in recent years because of advantages of better fixation system, better achievement of the target correction angle, and preservation of proximal tibiofibular joint [1–4].

However, HTO can only alter the proximal tibial geometry irrespective of the localization of the center of rotation and angulation of the lower limb. A previous study have noted that correcting only the proximal tibia with the aim of achieving a normal mechanical axis(MA) with HTO may result in overcorrection of the tibia [5]. An overcorrection in the proximal tibia may induce abnormal knee joint line orientation (KJLO), which has been raised as a concern in previous studies for the negative effect on joint biomechanics [6–9]. KJLO plays a crucial role in analysing the balance of forces across the knee joint. It represents the shear stress and biomechanical consequences of joint loading [10, 11]. A previous study has shown that KJLO of more than 5° induced excessive shear stress in the tibial articular cartilage [12].

A joint line parallel to the floor is an important objective after HTO [13]. However, no tools are currently available for surgeons that could predict the value of postoperative KJLO prior to the surgery. Postoperative KJLO can only be measured months after surgery when the patient is able to bear full weight and a standing full-limb anteroposterior (AP) radiograph can be performed.

The primary aim of this study was to determine the effects of various preoperative anatomical alignment parameters to the postoperative KJLO. Based upon this analysis, we aimed to devise an equation that predicts the value of postoperative KJLO.

## Materials and methods

### Study subjects

A total of 59 consecutive patients (73 knees) who underwent OWHTO by a single surgeon between December 2012 and May 2016 were analysed retrospectively. Inclusion criteria were patients who underwent OWHTO because of symptomatic varus knee OA with follow-up radiographs at 1 year after OWHTO. The exclusion criteria were as follows: (1) HTO performed concomitantly with ligamentous surgery such as anterior cruciate ligament reconstruction (2) suboptimal radiographic quality.

**Fig. 1:**
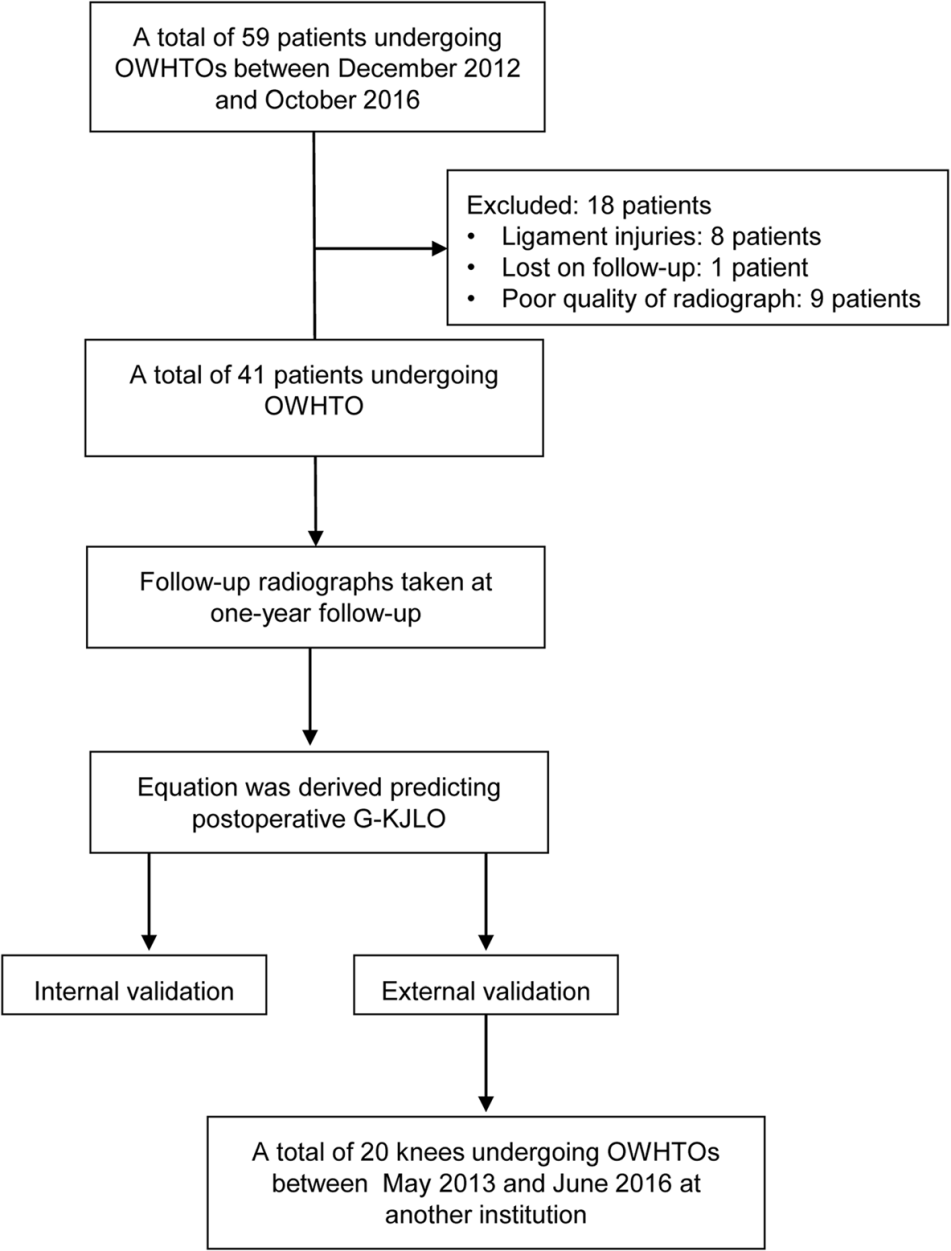
Flowchart of patient enrolment. OWHTO, open-wedge high tibial osteotomy; OA, osteoarthritis; G-KJLO, knee joint line orientation relative to the ground

A total of 18 patients (23 knees) were excluded: 8 patients who underwent concomitant ligamentous surgery, 9 patients whose postoperative radiographs were not optimal for full evaluation, and 1 patient who was lost in follow-up. Finally, 41 eligible patients (50 knees) were enrolled (Fig. 1). A total of 20 patients who underwent OWHTO between May 2013 and June 2016 at another institution by a different surgeon were evaluated for external validation of the equation.

### Surgical technique

Preoperative planning was done using a picture archiving and communication system (PACS). The aimed mechanical axis was the weight bearing line passing 62.5% of the width of the tibial plateau. The consequent correction angle to acquire the target mechanical axis were evaluated with the Miniaci method. A vertical skin incision was made between the anterior border of the medial collateral ligament medial border and the medial border of the patellar tendon. Two Steinmann pins were first inserted from the meta-diaphyseal junction toward the fibular tip. The initial osteotomy was performed tangent to these pins. A second coronal osteotomy was performed posterior to the tibial tubercle. The gap was gradually expanded until the target mechanical axis was achieved. Surgical site was then secured with a locking plate (Tomofix; Synthes). Cancellous bone chips were used to fill the osteotomy gap. All patients followed the same rehabilitation protocol. Patients started continuous passive movement exercises 2 days after the surgery. Patients were instructed to maintain partial weight bearing with crutch-assistance for 6 weeks and were allowed full weight bearing afterwards.

### Radiographic evaluation

In this study, 14 radiographic parameters, including (1) preoperative and postoperative knee joint line orientation relative to the ground (G-KJLO), (2) preoperative and postoperative ankle joint line orientation relative to the ground (G-AJLO), (3) preoperative and postoperative tibia plateau inclination (TPI), (4) preoperative and postoperative mechanical tibiofemoral angle (MTFA), (5) tibial length, (6) tibial width, (7) femoral condylar orientation (FCO), (8) joint space tilt angle (JSTA), (9) femoral bowing angle (FBA), and (10) correction angle, were evaluated with standing full-limb AP radiographs taken pre- and postoperatively. A reference foot template was used to control the foot rotation angle thus managing the rotational position of the radiograph. A patellar facing forward position was confirmed before the acquisition of full-limb AP radiograph [14]. Assessment of radiographs was performed using PACS (Maroview, Seoul, Korea).

**Fig. 2:**
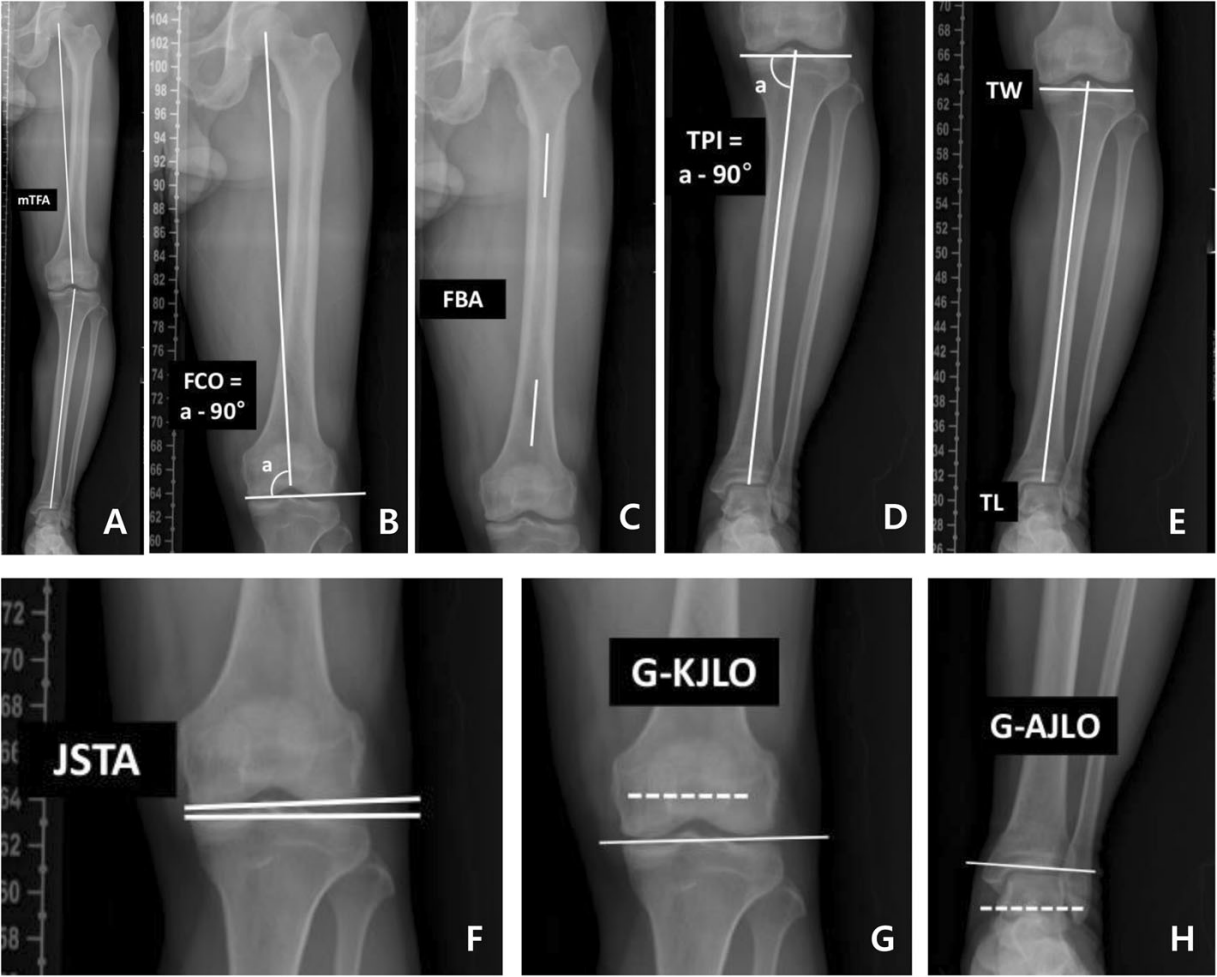
Radiographic parameters. **a** Mechanical tibiofemoral angle (MTFA), solid lines; **b** femoral condylar orientation (FCO), solid lines; **c** femoral bowing angle (FBA), solid lines; **d** tibial plateau inclination (TPI), sold lines; **e** tibial width (TW) and tibial length (TL), solid lines; **f** joint space tilt angle (JSTA), solid lines; **g** knee joint line orientation relative to the ground (G-KJLO), solid line; and **h** ankle joint line orientation relative to the ground (G-AJLO), solid line. Dotted line indicates the orientation of the ground

MTFA was defined as the angle formed by the intersection of the mechanical axis of the femur (the line from the femoral head centre to the femoral intercondylar notch centre) with the tibia (the line from ankle talus centre to the centre of the tibial spine tips); the knee in varus was given a negative value (Fig. 2a) [15]. FCO was defined as the angle between the mechanical axis of the femur and the tangent to the subchondral plates of both femoral condyles; a negative value was given in varus orientation (Fig. 2b) [16]. FBA was defined as the angle between the line connecting the points bisecting the femur at 0 and 5 cm below the lowest portion of the lesser trochanter and the line connecting the points bisecting the femur at 5 cm and 10 cm above the lowest portion of the lateral femoral condyle; a positive value was given to subjects with lateral bowing (Fig. 2c) [17]. TPI was defined as the angle between the mechanical axis of the tibia and the tangent to the subchondral plate of the tibia; a negative value was given in varus orientation (Fig. 2d) [8]. The tibial length was defined as the distance between the central point of the tibial spine and the central point of the tibial plafond surface [17]. The tibial width was defined as the distance between the lateral and medial end of the subchondral plate of the proximal tibia (Fig. 2e) [17]. JSTA was defined as the angle between the tangent to the subchondral plates of both femoral condyles and the tangent to the subchondral plate of the tibia; a negative value was given to the knee in more lateral space opening (Fig. 2f) [18]. The angle between the horizontal line parallel to the ground and the line bisecting the midpoints of lateral and medial joint space was defined as G-KJLO; bisecting line of the joint space tilted medially was given a negative value (Fig. 2g) [8]. The angle between the horizontal line parallel to the ground and the line tangent to the talus surface was defined as G-AJLO; tangential line to the talus surface tilted medially was given a negative value (Fig. 2h) [19]. The correction angle was defined as the value derived from subtraction of preoperative TPI from postoperative TPI.

### Statistical analysis

All the statistical analyses were carried out with IBM SPSS Statistics for Windows version 20.0 (IBM Corp., USA) and R version 3.4.2 (http://www.r-project.org). Significance was defined as a *P*-value < 0.05. The changes of the 4 radiographic parameters before and after HTO were compared using the paired t-test. Various factors related with the postoperative G-KJLO were analysed with the use of univariable and multivariable linear regression analyses. To avoid multicollinearity problem, stepwise selection was applied in the multivariable linear regression. The result of the final model was presented with β-coefficients, *t*-values, *P*-values, and adjusted coefficients of determination (*R*_*adj*_^*2*^). The model was internally and externally validated for calibration using bootstrapping resampling method with 1000 replicates. Furthermore, predicted squared correlation coefficient was calculated for external validity.

We estimated a sample size that could detect 2° difference in the mean postoperative G-KJLO which was considered clinically meaningful with the use of independent t-test. Based on the data from a prior study [5], at least 46 knees were needed to distinguish this difference. The type 1 error was 0.05 and the power was 0.8. Thus, this estimation would prove appropriateness.

To regulate intraobserver and interobserver agreement of radiologic evaluation, two orthopaedic surgeons evaluated radiologic measurements in 30 selected cases twice, with a three-week period between measurements. The intraobserver and interobserver agreement of evaluations for the 14 radiologic parameters were assessed with intraclass correlation coefficients. Intraclass correlation coefficients of intraobserver and interobserver agreement of radiologic evaluations were acceptable, > 0.89 (range, 0.89–0.99); evaluation from one author was used in the final analysis.

This study was approved by the institutional review board of our hospital.

## Results

This group included 36 women and 5 men with a mean age of 55 years (standard deviation [SD] 4.8; range 39–64) and a mean body mass index (BMI) of 26.1 kg/m^2^ (SD 3.2; range, 20.3–32.7) (Table 1).

**Table 1:** Baseline characteristics of the study groups

Clinical characteristics	Derivation set (*n* = 50)		Validation set (*n* = 20)	
	Mean	SD (range)	Mean	SD (range)
Age (years)	55.5	4.9 (39 to 64)	57.6	4.3 (48 to 66)
Male (n)	5 (12.1%)		4(20%)	
BMI	26.1	3.2 (20.3 to 32.7)	27.3	2.6 (20.5 to 31.9)
Left operation (n)	30 (60%)		12 (60%)	
Preoperative G-KJLO (°)	− 0.8	2.4 (− 5.6 to 3.9)	− 3.3	2.63 (− 7.6 to 3.4)
Preoperative G-AJLO (°)	8.4	3.3 (0.8 to 15)	10.1	2.8 (3.1 to 12.1)
Preoperative TPI (°)	−5.7	2.0 (−10.5 to − 2.5)	−6.5	2.1 (− 9.8 to − 1.3)
Preoperative MTFA (°)	−7.7	2.1 (−12.9 to − 2.3)	−7.8	2.9 (−14 to − 2.6)
Preoperative TW (mm)	73.1	4.4 (66.7 to 86)	74.2	7.2 (65.9 to 89.4)
Preoperative TL (mm)	340.5	21.7 (296 to 392)	334.1	13.8 (308.1 to 349.4)
Preoperative FCO (°)	1.7	2.0 (−2.6 to 5.6)	− 0.01	2.6 (−5 to 4)
Preoperative JSTA (°)	−3.6	2.2 (−9.6 to − 0.1)	− 2.6	1.8 (−6 to 0.3)
Preoperative FBA (°)	−0.07	3.1 (−7.4 to 7.1)	1.37	2.3 (− 4.4 to 5.02)
Correction angle (°)^a^	9.1	2.1 (7.0 to 15.2)	9.5	3.7 (4.5 to 19.6)

*BMI* Body mass index ratio, *G-KJLO* Knee joint line orientation relative to the ground, *G-AJLO* Ankle joint line orientation relative to the ground, *TPI* Tibia plateau inclination, *MTFA* Mechanical tibiofemoral angle, *TW* Tibial width, *TL* Tibial length, *FCO* Femoral condylar orientation, *JSTA* Joint space tilting angle, *FBA* Femoral bowing angle, *SD* Standard deviation

^a^Correction angle was defined as the value derived from subtraction of preoperative TPI from postoperative TP

**Fig. 3:**
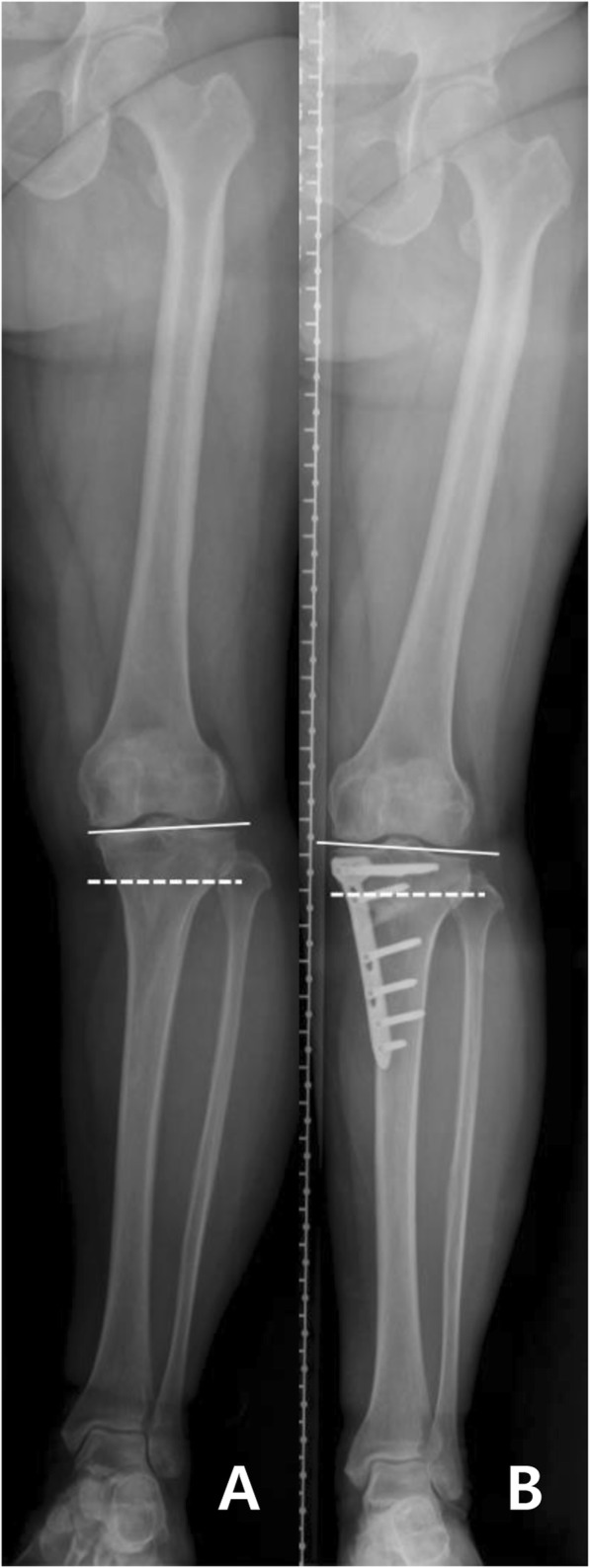
Radiographs showing changes of the G-KJLO after OWHTO. A patient underwent OWHTO due to varus OA. **a** Preoperative full-limb anterior-posterior radiograph of the patient. G-KJLO (solid line) was − 2.5° relative to the ground (dotted line). **b** Postoperative full-limb anterior-posterior radiograph of the patient. G-KJLO (solid line) was 2.6° relative to the ground (dotted line). OWHTO, open-wedge high tibial osteotomy; OA, osteoarthritis; G-KJLO, knee joint line orientation relative to the ground

MTFA, TPI, G-KJLO, and G-AJLO significantly changed after HTO. G-KJLO increased after HTO from a mean value of − 0.8° to 2.9° (*P* < 0.001). G-AJLO decreased after HTO from a mean value of 8.3° to 1.5° (*P* < 0.001) (Table 2 and Fig. 3).

**Table 2:** Comparative results of radiographic parameters before and after HTO

Parameter	Pre-HTO		Post-HTO		Mean difference (range)	*P*-value
	Mean	95% CI	Mean	95% CI		
MTFA	−7.7°	−9.8° to − 5.6°	2.4°	0° to 4.8°	10.2° (9.2° to 11.2°)	< 0.001
Tibia plateau inclination	−5.8°	−7.8° to −3.8°	3.3°	0° to 6.6 °	9.0° (8.1° to 10°)	< 0.001
G-KJLO	−0.8°	−3.2° to 1.6°	2.9°	0.3° to 5.5 °	3.8° (3.2° to 4.4°)	< 0.001
G-AJLO	8.3°	5° to 11.6°	1.5°	−2.2° to 5.2°	6.8° (6° to 7.6°)	< 0.001

*CI* Confidence interval, *MTFA* Mechanical tibiofemoral angle, *HTO* High tibial osteotomy, *G-KJLO* Knee joint line orientation relative to the ground, *G-AJLO* Ankle joint line orientation relative to the ground

Preoperative G-KJLO (*P* = 0.004), preoperative TPI (*P* = 0.001), preoperative MTFA (*P* = 0.004), preoperative tibial width (*P* = 0.011), preoperative FCO (*P* < 0.001), preoperative FBA, and correction angle (*P* < 0.001) were the most significant contributors to postoperative G-KJLO univariate analysis (Table 3).

**Table 3:** Univariable and Multivariable linear regression analyses of factors influencing postoperative G-KJLO after HTO in derivation set (*n* = 50)

	Univariable analysis			Multivariable analysis		
	β-coefficient	*t*-value	*P*-value	β-coefficient	*t*-value	*P*-value
Postoperative G-KJLO (°)						
BMI	−0.030	−0.206	0.838			
Preoperative G-KJLO (°)	0.673	3.069	< 0.001	0.560	5.882	< 0.001
Preoperative G-AJLO (°)	0.152	1.067	0.291			
Preoperative TPI (°)	0.318	2.322	0.025	0.310	2.574	0.013
Preoperative MTFA (°)	−0.398	−3.009	0.004			
Preoperative tibial width (mm)	−0.355	−2.628	0.011			
Preoperative tibial length (mm)	−0.256	−1.838	0.072			
Preoperative FCO (°)	−0.618	−5.447	< 0.001			
Preoperative JSTA (°)	−0.059	−0.412	0.682			
Preoperative FBA (°)	−0.440	−3.391	0.001			
Correction angle (°) ^a^	0.557	4.642	< 0.001	0.463	7.071	< 0.001

*BMI* Body mass index, *G-KJLO* Knee joint line orientation relative to the ground, *G-AJLO* Ankle joint line orientation relative to the ground, *TPI* Tibia plateau inclination, *MTFA* Mechanical tibiofemoral angle, *FCO* Femoral condylar orientation, *JSTA* Joint space tilting angle, *FBA* Femoral bowing angle, *HTO* High tibial osteotomy

^a^Correction angle was defined as the value derived from subtraction of preoperative TPI from postoperative TPI

Based on the multiple linear regression analysis by stepwise elimination of parameters influencing postoperative G-KJLO, an equation has been derived that can estimate postoperative G-KJLO after HTO. The equation is as follows: postoperative G-KJLO (°) = 1.029 + 0.560 × preoperative G-KJLO (°) + 0.310 × preoperative TPI (°) + 0.463 × aimed correction angle (°). The *R*_*adj*_^*2*^ value for this equation was 0.721.

**Fig. 4:**
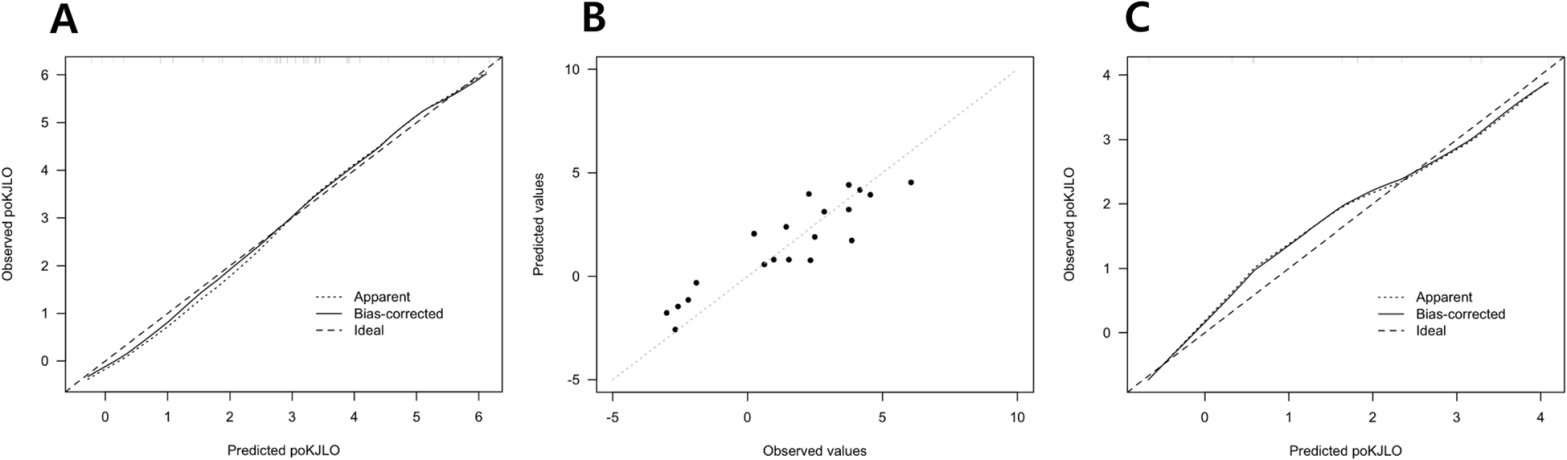
Internal and external validation of the equation. **a** Internal validation of the equation showing satisfactory performance of the equation. **b** and **c** External validation of the equation showing satisfactory performance of the equation poKJLO, postoperative knee joint line orientation relative to the ground

Internal validation using bootstrapping method showed good calibration performance of the equation (Fig. 4a). External validation also showed satisfactory performance of the equation with predicted squared correlation coefficient of 0.867 (Fig. 4b and c).

## Discussion

The most important finding in this study is the development of an equation that predicts the postoperative knee joint line orientation relative to the ground based on preoperative anatomical parameters measured from a single radiograph of the lower limb. The adjusted coefficients of determination value for this equation was 0.721.

Accurate preoperative planning is mandatory in the success of HTO. Several methods have been proposed by Hernigou et al. [2], Miniaci et al. [20], and Dugdale et al. [21] using a full-limb radiograph. These methods help the surgeons in determining the correction angle, preoperatively. Intraoperatively, Krettek et al. introduced the “cable method,” [22] and Lobenhoffer et al. used the “alignment rod method” to check the mechanical axis [23]. However, unexpected correction errors in HTO have been reported in many studies [24, 25]. The reason for these correction errors are unknown. Soft tissue laxity has been suspected as a factor affecting correction accuracy. Recently, one study has reported that joint JSTA can preoperatively quantify soft tissue laxity in HTO [26]. Our study also included JSTA in our analysis which would account for the soft tissue laxity. The postoperative value of G-KJLO, however, is unknown until a few months after the operation when the patient is strong enough to stand up straight to take a full-limb radiograph. Several cases have been reported where the G-KJLO is non-anatomical after HTO, while the mechanical axis is within normal range. Victor et al. reported that in knees of constitutional varus with advanced arthritis, KJLO is of a positive value because of the bone loss at the level of the medial distal femur [5]. Since HTO is an alignment procedure correcting only the proximal tibia, therefore, the bone loss at distal femur is not corrected. The aim to achieve normal mechanical axis with HTO, when anatomical factors contributing to varus alignment is still present such as in distal femur, results in overcorrection of the tibia which in turn increases the value of G-KJLO [27].

As shown in our multivariable linear regression analysis, several anatomical factors related to the postoperative G-KJLO are known. This represents that G-KJLO is not simply decided by a single factor but rather by a complex mixture of factors. One notable fact in our equation is that aimed correction angle is the only factor that can be controlled by the surgeon. Other factors are anatomical factors already decided before the surgery. It is a reasonable derivation to conclude that increasing 1° of correction angle in HTO increases 0.463° of G-KJLO. Preoperative G-KJLO, TPI, and aimed angle of correction were the most predictable factors associated with G-KJLO. In this study, 3 variables did not show any multicollinearity problems. Preoperative MTFA, preoperative tibial width, preoperative FCO, and preoperative FBA also showed to be significantly related to G-KJLO in univariable analysis; however, it was not included in the equation due to the correlation with TPI. The preoperative G-AJLO was expected to significantly affect the value of postoperative G-KJLO. Thus, it would have been included in our final equation because Lee et al. previously reported that G-KJLO changed significantly less than did the TPI after OWHTO because of the compensatory changes of G-AJLO [8]. The reason for elimination may be because G-AJLO is a dependent variable of G-KJLO.

**Fig. 5:**
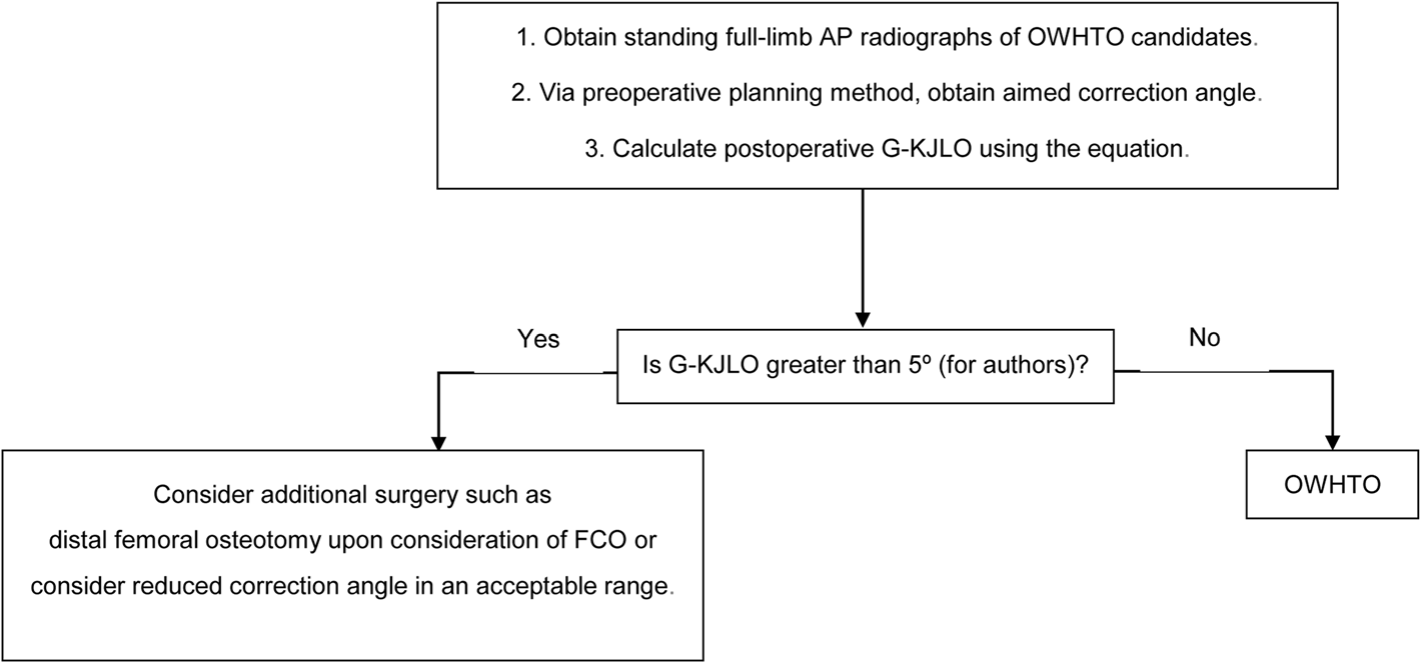
Algorithm in planning OWHTO. OWHTO, open-wedge high tibial osteotomy; AP, anteroposterior; G-KJLO, knee joint line orientation relative to the ground; FCO, femoral condylar orientation

With the use of our equation, the surgeon can estimate the postoperative value of G-KJLO, preoperatively. Predicting the G-KJLO prior to the surgery is very helpful to the surgeon because there are cases in which the postoperative G-KJLO is greater than a certain value while the mechanical axis is within normal range after HTO. As seen in our algorithm (Fig. 5), if the estimated postoperative G-KJLO is expected to be greater than a certain value, the surgeon should consider for an additional surgery, such as distal femoral osteotomy in addition to HTO or consider reducing the amount of correction. The most accepted target for the weight bearing line is 62.5% of the width of the tibia plateau. However, this value was based on the empirical results of Fujisawa et al. [28] Up to date, no clear scientific background of the correct target point is present. Birmingham et al. [29] proposed of templating the weight bearing line depending on the articular cartilage status of the lateral compartment. If the cartilage status of lateral compartment is of good quality, weight bearing line is templated towards the Fujisawa point. If the cartilage status of lateral compartment is unsatisfactory, weight bearing line is titrated towards the neutral point. Similarly, our algorithm suggests that in cases where G-KJLO is expected to be more than a certain value, the degree of correction should be reduced. The amount of correction, however, should not be reduced as to induce undercorrection of the malalignment. The weight bearing line should be titrated between neutral point and Fujisawa point. If the estimated postoperative G-KJLO is less than a certain value, the surgeon should undergo just the HTO as planned. Knowing the possibility of a secondary surgery beforehand is very helpful to the surgeon because there are a lot of factors influenced by an additional surgery, such as surgical time, surgical instruments, and tourniquet position. Informing the patient on the possibility of a secondary surgery beforehand is important. In our institution, we chose the certain value to be 5° based on the study of Nakayama et al. in which they stated that G-KJLO of more than 5° induced excessive shear stress in the tibial articular cartilage [12].

Some limitation should be noted in our study. First, this study only included patients with varus alignment who underwent OWHTO. The equation is not assured for the use in patients with valgus alignment who is planned for varus-producing osteotomy. Second, the anatomical alignment factors analysed in this study were measured only from standing full-limb AP radiograph. X-ray only reflects a two-dimensional aspect of a three-dimensional human structure. Therefore, predicting G-KJLO would have been more accurate if it was done with means of a three-dimensional imaging tool or gait analysis, even though it would have been less practical. We tried, however, to make the equation as simple and practical as possible, making it possible to derive G-KJLO with the use of only one AP radiograph of the lower limb.

## Conclusion

In this study, we analysed which preoperative anatomical alignment factors contributed to the postoperative G-KJLO. Based on this analysis, we were able to devise an equation that could predict postoperative G-KJLO with preoperative anatomical alignment factors. This equation is expected to help select optimal patients and operative plan for HTO.

## Data Availability

Data and materials can be accessed through a request to the lead author.

## Abbreviations

AP: Anteroposterior
FBA: Femoral bowing angle
FCO: Femoral condylar orientation
G-AJLO: Ankle joint line orientation relative to the ground
G-KJLO: Knee joint line orientation relative to the ground
HKA: Hip-knee-ankle
HTO: High tibial osteotomy
JSTA: Joint space tilt angle
LDFA: Lateral distal femoral angle
MPTA: Medial proximal tibial angle
OA: Osteoarthritis
OWHTO: Open wedge high tibial osteotomy
PACS: Picture archiving and communication system
TPI: Tibia plateau inclination

## References

[1] El-Azab H, Halawa A, Anetzberger H, Imhoff AB, Hinterwimmer S (2008). The effect of closed- and open-wedge high tibial osteotomy on tibial slope: a retrospective radiological review of 120 cases. J Bone Joint Surg Br.

[2] Hernigou P, Medevielle D, Debeyre J, Goutallier D (1987). Proximal tibial osteotomy for osteoarthritis with varus deformity. A ten to thirteen-year follow-up study. J Bone Joint Surg Am.

[3] Lee DC, Byun SJ (2012). High tibial osteotomy. Knee Surg Relat Res.

[4] Wang JH, Bae JH, Lim HC, Shon WY, Kim CW, Cho JW (2009). Medial open wedge high tibial osteotomy: the effect of the cortical hinge on posterior tibial slope. Am J Sports Med.

[5] Victor JM, Bassens D, Bellemans J, Gursu S, Dhollander AA, Verdonk PC (2014). Constitutional varus does not affect joint line orientation in the coronal plane. Clin Orthop Relat Res.

[6] Babis GC, An KN, Chao EY, Rand JA, Sim FH (2002). Double level osteotomy of the knee: a method to retain joint-line obliquity. Clinical results. J Bone Joint Surg Am.

[7] Hofmann S, Lobenhoffer P, Staubli A, Van Heerwaarden R (2009). Osteotomies of the knee joint in patients with monocompartmental arthritis. Der Orthopade.

[8] Lee KM, Chang CB, Park MS, Kang SB, Kim TK, Chung CY (2015). Changes of knee joint and ankle joint orientations after high tibial osteotomy. Osteoarthr Cartil.

[9] Oh K-J, Ko YB, Bae JH, Yoon ST, Kim JG (2016). Analysis of knee joint line obliquity after high tibial osteotomy. J Knee Surg.

[10] Amis AA (2013). Biomechanics of high tibial osteotomy. Knee Surg Sports Traumatol Arthrosc.

[11] Cooke TD, Pichora D, Siu D, Scudamore RA, Bryant JT (1989). Surgical implications of varus deformity of the knee with obliquity of joint surfaces. J Bone Joint Surg Br.

[12] Nakayama Hiroshi, Schröter Steffen, Yamamoto Chie, Iseki Tomoya, Kanto Ryo, Kurosaka Kenji, Kambara Shunichiro, Yoshiya Shinichi, Higa Masaru (2017). Large correction in opening wedge high tibial osteotomy with resultant joint-line obliquity induces excessive shear stress on the articular cartilage. Knee Surgery, Sports Traumatology, Arthroscopy.

[13] Thienpont E, Cornu O, Bellemans J, Victor J (2015). Current opinions about coronal plane alignment in total knee arthroplasty: a survey article. Acta Orthop Belg.

[14] Paley D, Herzenberg JE, Tetsworth K, McKie J, Bhave A (1994). Deformity planning for frontal and sagittal plane corrective osteotomies. Orthop Clin North Am.

[15] Hagstedt B, Norman O, Olsson TH, Tjornstrand B (1980). Technical accuracy in high tibial osteotomy for gonarthrosis. Acta Orthop Scand.

[16] van Raaij TM, Takacs I, Reijman M, Verhaar JA (2009). Varus inclination of the proximal tibia or the distal femur does not influence high tibial osteotomy outcome. Knee Surg Sports Traumatol Arthrosc.

[17] Nagamine R, Miura H, Bravo CV, Urabe K, Matsuda S, Miyanishi K, Hirata G, Iwamoto Y (2000). Anatomic variations should be considered in total knee arthroplasty. J Orthop Sci.

[18] Terauchi M, Shirakura K, Katayama M, Higuchi H, Takagishi K, Kimura M (2002). Varus inclination of the distal femur and high tibial osteotomy. J Bone Joint Surg Br.

[19] Lee JH, Jeong BO (2012). Radiologic changes of ankle joint after total knee arthroplasty. Foot Ankle Int.

[20] Miniaci A, Ballmer FT, Ballmer PM, Jakob RP. Proximal tibial osteotomy. A new fixation device. Clin Orthop Relat Res. 1989;(246):250–9. 2766613

[21] Dugdale TW, Noyes FR, Styer D. Preoperative planning for high tibial osteotomy. The effect of lateral tibiofemoral separation and tibiofemoral length. Clin Orthop Relat Res. 1992;(274):248–64. 1729010

[22] Krettek C, Miclau T, Grun O, Schandelmaier P, Tscherne H (1998). Intraoperative control of axes, rotation and length in femoral and tibial fractures. Technical note. Injury.

[23] Freiling D, van Heerwaarden R, Staubli A, Lobenhoffer P (2010). The medial closed-wedge osteotomy of the distal femur for the treatment of unicompartmental lateral osteoarthritis of the knee. Oper Orthop Traumatol.

[24] Gaasbeek RD, Nicolaas L, Rijnberg WJ, van Loon CJ, van Kampen A (2010). Correction accuracy and collateral laxity in open versus closed wedge high tibial osteotomy. A one-year randomised controlled study. Int Orthop.

[25] Marti CB, Gautier E, Wachtl SW, Jakob RP (2004). Accuracy of frontal and sagittal plane correction in open-wedge high tibial osteotomy. Arthroscopy.

[26] Lee DK, Wang JH, Won Y, Min YK, Jaiswal S, Lee BH, Kim JY. Preoperative latent medial laxity and correction angle are crucial factors for overcorrection in medial open-wedge high tibial osteotomy. Knee Surg Sports Traumatol Arthrosc. 2019. 10.1007/s00167-019-05502-6. 10.1007/s00167-019-05502-6 30980121

[27] Floerkemeier S, Staubli AE, Schroeter S, Goldhahn S, Lobenhoffer P (2013). Outcome after high tibial open-wedge osteotomy: a retrospective evaluation of 533 patients. Knee Surg Sports Traumatol Arthrosc.

[28] Fujisawa Y, Masuhara K, Shiomi S (1979). The effect of high tibial osteotomy on osteoarthritis of the knee. An arthroscopic study of 54 knee joints. Orthop Clin North Am.

[29] Birmingham TB, Giffin JR, Chesworth BM, Bryant DM, Litchfield RB, Willits K, Jenkyn TR, Fowler PJ (2009). Medial opening wedge high tibial osteotomy: a prospective cohort study of gait, radiographic, and patient-reported outcomes. Arthritis Rheum.

